# AC-NP: A Novel Chimeric Peptide with Natriuretic and Vasorelaxing Actions

**DOI:** 10.1371/journal.pone.0020477

**Published:** 2011-05-24

**Authors:** Bao-Ying Chen, Jian-Kang Chen, Miao-Zhang Zhu, Dong-Liang Zhang, Jie-Shen Sun, Jian-Ming Pei, Hua-Song Feng, Xiao-Xing Zhu, Jian Jin, Jun Yu

**Affiliations:** 1 Department of Radiology, Tangdu Hospital, Fourth Military Medical University, Xi'an, China; 2 School of Basic Medical Science, Center of Teaching Experiment, Fourth Military Medical University, Xi'an, China; 3 Department of Physiology, Fourth Military Medical University, Xi'an, China; 4 Department of Respiration Disease, General Hospital of Navy Force, Beijing, China; 5 Department of Pharmacology, Fourth Military Medical University, Xi'an, China; 6 Laboratory of Molecular Pharmacology, School of Medicine and Pharmaceutics, Jiangnan University, Wuxi, China; L' Istituto di Biomedicina ed Immunologia Molecolare, Consiglio Nazionale delle Ricerche, Italy

## Abstract

The aim of this study was to evaluate the cardiovascular and renal activities of a newly designed natriuretic peptide (NP). Here, we engineered a novel 28-amino acid chimeric peptide, termed AC-NP that combined the 17-amino acid ring of C type natriuretic peptide (CNP) with the 6-amino acid N-terminus and 5-amino acid C-terminus of atrial natriuretic peptide (ANP). Both in vitro and in vivo experiments were performed to determine the actions of AC-NP. In normal rats, AC-NP proved to be more potentially diuretic, natriuretic and hypotensive compared with other NPs, such as ANP, CNP and vasonatrin peptide (VNP), which is another man-made NP. In relaxation of isolated abdominal aorta from rat, AC-NP was equally effective to ANP, CNP and VNP. Elevated levels of 3′,5′-guanosine monophosphate (cGMP) in plasma and urine cGMP excretion indicated the participation of cGMP in the functions of AC-NP. Taken together, innovative designed AD-NP might be a new candidate therapeutic peptide against cardiorenal disorders.

## Introduction

Human natriuretic peptides (NPs) are a family of structurally similar but genetically distinct peptides including atrial natriuretic peptide (ANP), brain natriuretic peptide (BNP), and C type natriuretic peptide (CNP). NPs are known to play important roles in the regulation of cardiorenal homeostasis [1]. ANP is a 28-amino acid peptide, whereas BNP contains 32 amino acids, and CNP contains 22. All of them have a 17-amino acid ringed structure, which has been identified as essential for their pharmacological activity [2]. Most functions of NPs appear to be mediated through the elevation of intracellular cyclic 3′,5′-guanosine monophosphate (cGMP) after their binding to natriuretic peptide receptors (NPR), NPR-A and NPR-B, which are coupled to the particulate guanylyl cyclase. ANP and BNP mainly bind to NPR-A, while CNP functions via NPR-B [3].

Unlike ANP or BNP, CNP lacks a C-terminus amino acid extension, which might account for its weakness of natriuretic actions [4]–[6]. This limits the application of CNP against disorders with sodium-and water-retaining syndromes, such as acute heart failure (AHF) despite its attractive venodilating advantages [6], [7].To optimize the structure of natural NPs, strategy of proteins engineering was advanced: to fuses beneficial structural elements unique for one peptide with active structures from separate peptides to create chimeras, which possess attractive therapeutic properties of the parental molecules [8].

In 1993, Wei CM et al. [9] invented vasonatrin peptide (VNP), the man-made novel member of the NPs family. VNP is a chimera of CNP and ANP possessing the 22-amino acid ringed structure of CNP, along with the C-terminus of ANP. VNP has the venodilating actions of CNP, the natriuretic actions of ANP, and unique arterial vasodilating actions not associated with either ANP or CNP. In addition, Lisy O et al. [10] engineered the C-terminus of *Dendroaspis* natriuretic peptide (DNP) to the structure of CNP and got a novel NP, termed AD-NP. Demonstrated in vivo, CD-NP is natriuretic and diuretic, glomerular filtration rate enhancing, cardiac unloading, and renin inhibiting. However, DNP was originally isolated from the green mamba. Its non-human origin challenged the safety of its utility in human beings. Recently, Kilic A et al. [11] reported CU-NP, a non-vasodilatating novel NP synthesized from the ring structure of CNP and both C- and N-termini of urodilatin. CU-NP had direct anti-hypertrophic effect on cultured neonatal rat ventricular myocytes exposed to phenylephrine. Urodilatin is a 32-amino acid peptide generated from alternative processing of ANP expressed in the kidney [12]. Inspire by the success of previous reported chimeric NPs, we designed an AC-NP, comprising the ring structure of CNP along with C- and N-termini of ANP, and identified its functional actions.

## Methods

### Reagents

**Figure 1:**
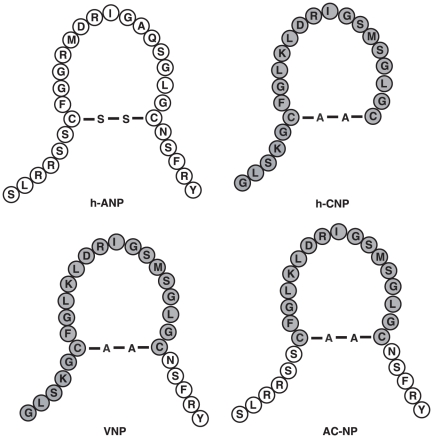
Amino acid sequences and structures of human ANP (h-ANP), human CNP (h-CNP), chimeric VNP and novel chimeric AC-NP.

Figure 1 illustrates the structures of ANP, CNP, VNP, and the new peptide, AC-NP. AC-NP is 28-amino acid in length including 17-amino acid ringed structure from CNP, as well as both C- and N-termini of ANP. ANP, CNP, VNP and AC-NP were all synthesized by School of Medicine and Pharmaceutics, Jiangnan University (Wuxi, China). L-norepinephrine bitartrate (NE) and acetylcholine chloride (Ach) were purchased from Sigma Chemical Co. (St. Louis, MO, USA). [^125^I]-cGMP assay kit was from Shanghai Chinese Medical University (Shanghai, China).

### Animals

Animal experiments were approved by University Ethics Committee (the Ethical Committee of Fourth Military Medical University on research with approval ID 2010-jxsyzx-1). Prior to the experiments, male Sprague-Dawley (SD) rats (250–350 g body weight; 6–8 weeks old) had been reared at an ambient temperature of 23±1°C and a relative humidity of 60–70%, with light exposure daily from 6:00 to 18:00 h. Each animal received a standard laboratory diet and tap water ad libitum throughout the experimental period.

### Arterial Ring Preparation and Vasoreactivity Measurement

Rings cut from abdominal aorta obtained from male SD rats (anesthetized with 40 mg/kg sodium pentobarbital intra peritonealy) were suspended for the measurement of isometric force in organ chambers filled with Krebs solution, maintained at 37°C and bubbled with a gas mixture of 95% O_2_-5% CO_2_ [13].The Krebs solution contained (in 10^−3^ M) 115 NaCl, 4.7 KCl, 25 NaHCO_3_, 1.2 MgCl_2_, 1.2 KH_2_PO_4_, 2.5 CaCl_2_, and 10 glucose; pH 7.4. In one-half of the rings, the endothelium was removed by gently rubbing the intimal surface with a cotton swab wet with control solution. For each individual vessel ring, the proper length-tension relationship during repeated exposures to 10^−1^ M KCl was determined by 100-mg increments until an optimal resting force around 500 mg was identified. All the subsequent pharmacological examinations were conducted at this initial resting force.

Experimental procedure is as follows. After 30 min of equilibration, the presence of functional endothelium was determined at the beginning of the experiment by relaxation to Ach (10^−6^ M) during a contraction to NE (10^−6^ M) at optimal length. In order to study the vasorelaxant properties of ANP, CNP, VNP or AC-NP, the artery segments were initially contracted with NE (10^−6^ M), which elicited 80% of maximal contraction. When the contraction reached a steady state, cumulative concentration-response curves to ANP, CNP, VNP or AC-NP were obtained by adding increasing logarithmic molar concentrations. The maximal contraction induced by 10^−6^ M extracellular NE was arbitrarily set as 100%. Relaxation produced by AC-NP, ANP, CNP or VNP was measured and expressed as the reduced percentages of maximal contractile amplitudes to 10^−6^ M NE.

### Examination of cardiovascular actions of AC-NP

Experiments were conducted in four groups (n = 6): ANP, CNP, VNP or AC-NP. SD rats were anesthetized with 40 mg/kg sodium pentobarbital intra peritonealy. Intravenous infusions of saline solution (0.9% NaCl) were performed (1 ml/100 gm body weight per hour) through the left jugular vein catheter. After completion of surgery, rats were allowed to stabilize for 30 min. In each group a 15 min baseline period followed. After the baseline period, the NPs (ANP, CNP, VNP, AC-NP) at 50 µg/kg or saline vehicle (0.9% NaCl) was administered in a bolus fashion (0.1 ml) and was followed by a 15-min period. After a 30-min washout, a 15-min recovery period followed.

During each experimental period, carotid arterial pressure (mCAP) was recorded via left carotid artery cannulation by the BL-420F BioData Acquisition & Analysis Systems (TME Technology Co, Ltd, Chengdu, China). At the midpoint of baseline and bolus, blood was sampled for analysis of plasma cGMP levels (PcGMP). At the end of baseline, and bolus period, urine collected for determination of urine volume (UV), urine sodium excretion (UNaV, by zeeman atomic absorption spectroscopy) and urine cGMP excretion (UcGMPV).

Blood for plasma cGMP analysis was collected into EDTA tubes, immediately placed on ice, and centrifuged at 2,500 rpm at 4°C. Plasma was separated and stored at −20°C until assay. Urine for cGMP determination was heated to over 90°C before storage. The levels of cGMP were determined by radioimmunoassay performed with a [^125^I]-cGMP assay kit previously described [14]. The sensitivities of the cGMP radioimmunoassay were 50 fmol per assay tube.

### Statistical Analysis

Data are expressed as the mean±SEM. Significant differences between the means were determined by one-way analysis of variance (ANOVA). If significant, group means were compared using Least significant difference (LSD) methods for multiple comparisons of means. Statistical significance was determined by a *P*<0.05, calculated by SPSS, version 13.0 (SPSS Science, Chicago, IL, USA).

## Results

As shown in Figure 1, both AC-NP and VNP are the chimeras of ANP and CNP. Structurally, ANP and CNP are the “parents” of AC-NP, whereas VNP and AC-NP are “brothers”. Allowing for the structural similarity of AC-NP, ANP, CNP and VNP, we therefore evaluated the actions of AC-NP compared with ANP, CNP and VNP.

### In Vitro Vasorelaxing Actions of AC-NP in Isolated Abdominal Aorta from Rat

**Figure 2:**
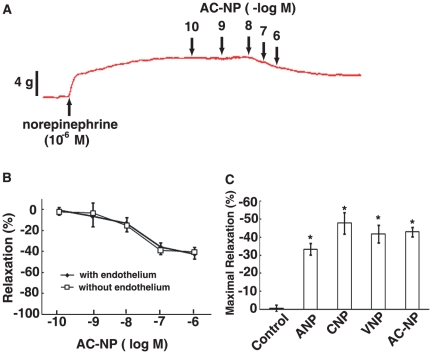
Relaxant responses of isolated abdominal aorta from rat to ANP, CNP, VNP or AC-NP. Endothelium-intact rings cut from abdominal aorta of 5 rats (n = 5) were suspended in organ chambers. In one-half of the rings, the endothelium was removed by gently rubbing the intimal surface with a cotton swab. After setting resting force to about 500 mg weight, artery rings were contracted with 10^−6^ M extracellular norepinephrine (NE) and relaxed with ANP, CNP, VNP or AC-NP. (A) The representative curves of relaxation to cumulatively increasing concentration of AC-NP. (B) Dose dependent response of isolated rat abdominal aorta with or without endothelium to cumulatively increasing concentration of AC-NP. (C) Maximal responses to ANP, CNP, VNP and AC-NP at the dose of 10^−6^ M, or saline as control. The maximal contraction induced by 10^−6^ M extracellular NE was arbitrarily set as 100%. Data of relaxation are the reduced (minus) percentages of maximal contractile amplitudes to 10^−6^ M NE, and presented as mean ± SEM. Symbols obscure some of the smaller error bars.

In the representative concentration-response curves illustrated in Figure 2A, AC-NP reduced the contractile responses of the arterial ring to 10^−6^ M NE in a dose dependent manner. The equivalent responses of deendothelium and endothelium-intact artery rings to AC-NP indicated that vasodilating actions of AC-NP are independent of endothelium (Fig. 2B). The maximal relaxations induced by ANP, CNP, VNP or AC-NP in isolated rat abdominal aorta were shown in Figure 2C. ANP, CNP, VNP or AC-NP at the dose of 10^−6^ M all caused significant relaxations compared with saline control (*P*<0.05, n = 5). The potency for relaxation in isolated rat abdominal aorta for the peptides was shown as: CNP > AC-NP > VNP > ANP, but not statistically significant.

### In Vivo Cardiorenal Actions of AC-NP in Rats

**Figure 3:**
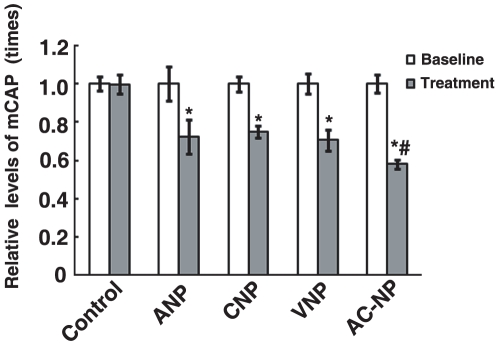
Effect of AC-NP on mean carotid arterial pressure (mCAP) in rats. The mCAP values were normalized against the corresponding values of baseline, which were arbitrarily set as 1. Data are expressed as means ± SEM, n = 6. *P<0.05 versus baseline. # *P*<0.05 versus ANP, CNP, or VNP group. Saline was used as control.

In accordance with the in vitro evidences, ANP, CNP, VNP or AC-NP at the dose of 10^−6^ M all induced significant decrease of mCAP in comparison with the level of baseline (*P*<0.05, n = 6). The decreased percentage of ANP, CNP, VNP and AC-NP was 24%, 15%, 29% and 44% indicating potential hypotensive effects of AC-NP (Fig. 3).

Also we observed potent natriuretic and diuretic responses to AC-NP in normal rats (Table 1). ANP, VNP and AC-NP all markedly enhanced the UV and UNa, in contrast to the non-obvious effects of CNP (*P*<0.05, n = 6). Importantly, AC-NP caused greater responses of both urine volume and urine sodium excretion compared with ANP and VNP. Thus, the natriuretic and diuretic responses to NPs were summarized as: AC-NP > ANP > VNP > CNP.

**Table 1:** Effects of AC-NP, ANP, CNP and VNP on UV and UNaV after intravenous injection in anesthetized rats (n = 6, mean ± *SEM*).

Natriuretic peptides (50 µg/kg)		UV (µl/min)	UNaV (µmol/min)
ANP	Baseline	7.4±1.1	0.6±0.1
	Treatment	28.9±2.2*	2.6±0.4*
CNP	Baseline	7.8±0.9	0.8±0.2
	Treatment	7.4±0.8	0.8±0.1
VNP	Baseline	8.7±1.2	0.9±0.2
	Treatment	19.2±1.8*	2.5±0.3*
AC-NP	Baseline	7.5±0.9	0.7±0.1
	Treatment	75±11.4*,#	7.1±0.7*,#

Note: UV, urine volume; UNaV, urine sodium excretion.

*P<0.01 versus the baseline levels.

# *P*<0.05 versus the levels of ANP, CNP or VNP-treated group.

**Figure 4:**
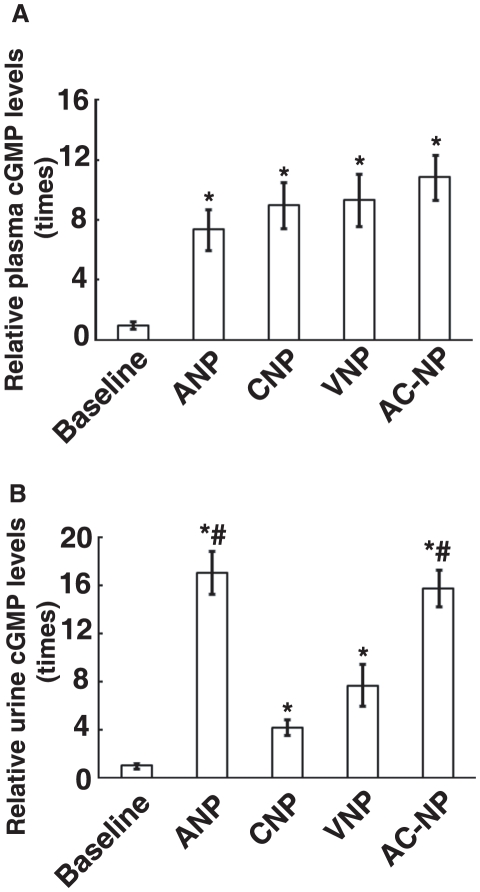
Responses of plasma cGMP levels (PcGMP) and urine cGMP excretion (UcGMPV) to ANP, CNP, VNP and AC-NP. Rats were treated with ANP, CNP, VNP or AC-NP (50 µg/kg). The levels of cGMP in plasma (A) or urine cGMP excretion (B) were determined by radioimmunity assay. The cGMP levels of baseline group were arbitrarily set as 1. Data represent mean±SEM, n = 6. * *P*<0.05 versus baseline, # *P*<0.05 versus CNP or VNP group.

NPRA/B/cGMP/PKG is the classical signaling pathway participating the bioactivities of NPs in cardiovascular and renal system [3]. Therefore, the levels of cGMP in urine as well as in the plasma were detected after treatment of rats with NPs. As shown in Figure 4, the enhancing effects on the levels of plasma cGMP induced by AC-NP were greatest among the NPs tested in current study. For urine cGMP excretion, the potency of AC-NP was significantly stronger than that of CNP or VNP (*P*<0.05, n = 6).

## Discussion

This study reports for the first time the cardiorenal actions of a new chimera derived from ANP and CNP. Termed AC-NP, the novel NP represents the 17-amino acid ringed structure of CNP along with the C- and N-termini of ANP. Functionally, AC-NP proved to be a potent vasorelaxing peptide similar to ANP, CNP and VNP. Specifically, AC-NP was more potently natriuretic and diuretic compared with ANP and VNP, contrast to the reported lack of significant renal actions of CNP [4], [6], [7]. The diuretic activity of AC-NP might contribute to its hypotensive effects.

The structural difference between ANP and CNP (Fig. 1) is in amino acid sequences and an absence of a C-terminus for CNP as noted for ANP. As for AC-NP, its C-terminus was from ANP. In a similar way, another chimeric NP, VNP, also adds C-terminus of ANP to the ringed structure of CNP. However, the N-terminus of VNP was that of CNP rather that of ANP. This is the structural difference between AC-NP and VNP. Our current work (Fig. 4) and previous reports on VNP [9] give evidences that addition of C-terminus to the ring of CNP results in gain of natriuretic functions. Importantly, engineering of termini of ANP and ring of CNP in AC-NP supports the conclusion that both C- and N-termini might play a functionally important role for biological activity of NPs. As a result, chimeric NPs including ACP-NP, VNP [9], and recently reported AD-NP [10], CU-NP [11] gain beneficial properties in comparison with their parental NPs. It suggested that engineering of chimeric NPs is a feasible strategy to optimize the structure of NPs for the sake of clinical applications.

In current study, we identified some cardiorenal actions of AC-NP. However, the mechanism of this new NP is largely unknown. Since cGMP is the classical second messenger of NPs-NPR-cGMP-PKG signaling pathway [3], AC-NP-induced elevation of plasma and urine cGMP levels suggests participating roles of cGMP in the action of AC-NP. It is known that ANP and BNP mainly bind to NPR-A, while CNP functions via NPR-B [2]. As the chimera of ANP and CNP, AC-NP possibly plays roles via NPR-A or NPR-B. It was reported that functional receptor subtypes for VNP, another chimera of ANP and CNP, in renal glomeruli and arteries was NPR-A [15]. Allowing for the homology between VNP and AC-NP, AC-NP is likely to act on NPR-A. However, our present study could not exclude the possibility of the participation of NPR-B in the function of AC-NP, further studies are required to give direct evidence.

In summary, we reported the design, synthesis, and cardiorenal actions AC-NP, a novel man-made NP, which represents a fusion peptide of the full-length 22-amino acid structure of CNP together with both C- and N-termini of ANP. Specifically, AC-NP demonstrated vasorelaxing, natriuretic and diuretic advantages compared with previously reported ANP, CNP and VNP. The properties of AC-NP especially suggests its possible usefulness against cardiorenal disorders with sodium-and water-retaining such as acute heart failure and acute myocardial infarction. Further intensive investigations of AC-NP in experimental models of cardiorenal disease and ultimately in human disease states will answer the question of whether this new NP will indeed act as efficacious and safe drugs.
